# Site-specific DNA double-strand breaks greatly increase stable transformation efficiency in *Trypanosoma brucei*

**DOI:** 10.1016/j.molbiopara.2009.03.010

**Published:** 2009-08

**Authors:** Lucy Glover, David Horn

**Affiliations:** London School of Hygiene & Tropical Medicine, Keppel Street, London, WC1E 7HT, UK

**Keywords:** I-SceI, Recombination, Repair

## Abstract

Genetic manipulation in African trypanosomes typically relies upon electroporation with chromosomal integration of DNA constructs by homologous recombination. Relatively little is known about chromosomal recombination and repair in these organisms however and low transformation efficiency and position effects can limit forward genetic approaches. In yeast and mammalian cells, site-specific DNA double-strand breaks (DSBs) stimulate targeted integration through homologous recombination-based repair where the exogenous DNA serves as the template. We have explored the effect of DSBs on targeted integration in bloodstream-form *Trypanosoma brucei*, focusing on the ribosomal RNA-spacer target commonly used to integrate recombinant constructs. DSB-repair within the ribosomal RNA tandem gene-repeats is likely dominated by single-strand annealing allowing approximately 80% of cells to survive the break. In the presence of exogenous DNA, transformation efficiency is increased approximately 250-fold by DSB-induction. In the example presented, more than 1% of cells that survive the procedure were transformed generating 80,000 transformants from a typical experiment.

Chromosomal double-strand break (DSB) repair mechanisms are crucial for genome stability and cell survival in all living organisms. Single DSBs in the core of *Trypanosoma brucei* chromosomes have been shown to trigger a robust DNA damage response and efficient repair via homologous recombination with allelic templates [1]. In yeast [2] and mammalian cells [3,4], homologous sequence on exogenously introduced DNA can also be used for repair such that DSBs stimulate stable chromosomal integration. The ability to manipulate trypanosomatid genomes [5,6] revolutionized molecular biology studies in these organisms and electroporation using the Nucleofector device and ‘T-cell’ solution (Lonza) now allows efficient stable transformation of bloodstream-form *T. brucei* [7]. However, forward genetic approaches are still limited by transformation efficiency and position effects, major differences in expression dependent upon the locus of integration.

With few loci shown to be transcriptionally inactive in trypanosomatids, the non-transcribed ribosomal RNA (*RRNA*) spacer loci [8] are probably the most popular targets for the integration of regulated transgenes. There are nine of these annotated in the haploid genome sequence (*T. brucei* is diploid) with one complete *RRNA* unit each on chromosomes 1 and 7, two on chromosome 2 and five on chromosome 3 [9] and position effects have been demonstrated when targeting the spacers at these loci [10]. I-SceI is a site-specific double-strand meganuclease that recognizes and cleaves an 18 bp sequence. I-SceI has been used previously to efficiently induce DSBs at specific loci on *T. brucei* chromosomes [1] with no evidence of non-specific toxicity [11]. Here, we have employed tetracycline-inducible I-SceI expression to introduce a specific DSB at a *RRNA*-spacer, to explore repair at this locus and to determine the effect on transformation efficiency.

Initially, to determine whether a DSB can increase transformation efficiency in *T. brucei*, we induced a DSB at a ‘single-copy’ gene locus on chromosome 11 where we had previously integrated a I-SceI site [1]. We added tetracycline to induce a DSB 0, 3 or 18 h prior to electroporation (BioRad, GenePulser II). The number of stably transformed cells was increased in all three samples exposed to tetracycline and peaked in the 3 h samples which generated 3 × 10^−5^ transformants, at least 300-fold more than control cells (Table 1, expt. 1). Similar results were obtained when we induced a DSB in the tandem tubulin gene array on chromosome 1 (Table 1, expt. 2).

Using standard electroporation, circular (uncut) and linear DNA are taken up by cells equally well but circular DNA displays reduced transformation efficiency [12,13], typically <10^−7^ in bloodstream-form cells (data not shown). We next examined how genomic breaks and exogenous DNA linearization combine to affect transformation. Circular exogenous DNA combined with 3 h, I-SceI-induced samples yielded 2 × 10^−6^ transformants while linear DNA yielded 7 × 10^−5^ transformants (Table 1, expt. 3). This indicated that both exogenous DNA linearization and genomic breaks promote stable integration in a cooperative manner, presumably because breaks facilitate strand-invasion [14]. We now proceeded to study recombination at *RRNA* loci.

For these studies, a Red Fluorescent Protein (*RFP*) – Puromycin *N*-ACetyltransferase (*PAC*) fusion gene with an embedded I-SceI site (*R*^*s*^*P*) and driven by an *RRNA* promoter, was assembled and targeted to *RRNA*-spacers in cells with a conditional I-SceI gene (Fig. 1A). RNA polymerase I transcription stimulates homologous recombination in *T. brucei* [15] and *RRNA*-spacer loci exert position effects on integrated promoters [10] so we screened several *R*^*s*^*P*_RRNA_ clones for direct RFP fluorescence and selected one with the highest expression to ensure robust transcription at the target site. The vast majority (>99%) of these *R*^*s*^*P*_RRNA_ cells revert to puromycin sensitivity when grown in tetracycline (data not shown) indicating efficient I-SceI cleavage and disruption or loss of the *R*^*s*^*P* cassette (see Fig. 1A). *R*^*s*^*P*_RRNA_ cells were then tested for transformation efficiency by inducing I-SceI expression 3 h prior to electroporation. This generated 8 × 10^−5^ transformants, 200-fold more than control cells (Table 1, expt. 4) and similar to the efficiency obtained when these conditions were used to target loci on chromosomes 1 and 11.

All *RRNA*-spacers, possibly excepting the one found on chromosome 1, are flanked by large stretches of duplicated sequence which would be expected to facilitate single-strand annealing. This form of repair can occur when a DSB is flanked by two related sequences; DNA resection reveals homologous sequences that anneal and facilitate repair with loss of the intervening DNA [14]. First, a clonogenic assay with a pair of independent *R*^*s*^*P*_RRNA_ strains demonstrated that ∼80% of cells survived the introduction of a DSB (Fig. 1B) and suggested that the targeted *RRNA*-spacer loci were efficiently repaired. We next used hybridization analysis to monitor the DNA-damage-response triggered by a DSB at a *RRNA*-spacer (in the absence of exogenous DNA). This demonstrated formation of single-stranded DNA adjacent to the break that peaked 12–24 h after induction and also loss of the *RFP* gene in the majority of induced cells after 48 h (Fig. 1C). We looked for features associated with allelic homologous recombination [1] but detected only a modest increase in G_2_M cells and RAD51 foci in ∼5% of cells (data not shown). Although more work is required to define the repair pathways employed, the results are consistent with predominant repair via single-strand annealing, as expected. A RAD51-dependent pathway, possibly allelic homologous recombination, may operate in ∼5% of cells.

We had chosen the BioRad Gene Pulser for our initial electroporation studies (above) because *T. brucei* chromosomal integration has been extensively characterized using this approach [16]. However, one goal was to combine gains in transformation efficiency from Nucleofection and DSB-repair. We considered this to be conceivable since Nucleofection is thought to increase efficiency by delivering DNA to the nucleus more efficiently than other electroporation devices and solutions (http://www.lonza.com/). Since Nucleofector buffer composition is proprietary, we decided to carry out some preliminary analyses. In particular, we wanted to determine whether Nucleofection was similarly dependent upon terminal homologous sequence on the exogenous DNA [12,13]. For this, we took advantage of the 2T1 system which requires a defined recombination event to generate drug-resistant cells [17]. Using exogenous DNA that was either circular or linear with internal or terminal targeting sequences, we obtained <1 × 10^−7^, 3 × 10^−7^ and 1 × 10^−5^ transformants respectively (Table 1, expt. 5). These results indicate that, as with standard electroporation, terminal targeting sequences on the exogenous DNA greatly increase integration efficiency. Nucleofection yields approximately 10^−4^ transformants when the tandem procyclin gene loci on chromosomes 6 and 10 are targeted [7], but we have seen locus-dependent differences in efficiency using this approach; we obtained approximately 10^−5^ transformants when targeting the full set of *RRNA*-spacer loci for example (data not shown).

We now proceeded to use Nucleofection to assess meganuclease-facilitated integration in *R*^*s*^*P*_RRNA_ cells (Fig. 2A). Without DSB-induction we obtained 1.2 × 10^−5^ transformants and with DSB-induction we obtained 3.2 × 10^−3^ transformants (Fig. 2B and Table 1, expt. 6). Thus, in the presence of a DSB, transfection efficiency is increased >250-fold and >1% of cells that survive the procedure are transformed. High efficiency, site-specific integration could be exploited to develop cost-effective and practical forward genetic approaches that minimise position effects.

## Figures and Tables

**Fig. 1:**
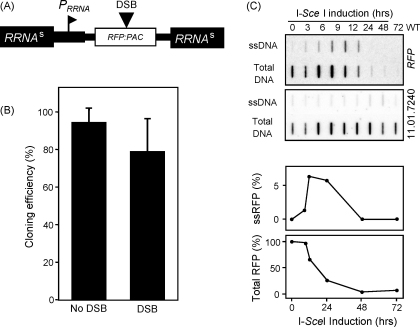
Response to a DSB at the *RRNA*-spacer locus. (A) The schematic map illustrates an *RFP–PAC* fusion gene (*R*^*s*^*P*) with an embedded I-SceI site (indicated by DSB) at the *RRNA*-spacer (*RRNA*^*s*^) locus. *pR*^*s*^*P*_RRNA_ was assembled as follows: an *RRNA* promoter (*P*_*RRNA*_) fragment was amplified from genomic DNA using primers RpF (GATCcggcggTAGCTTTCCACCCAGCGC) and RpR (GATCcggccgggcccACTGggatccTCTGAGAGCGGTCAGTTGC), digested with EagI (relevant restriction sites in lower-case) and ligated to a NotI-digested *RRNA*-spacer fragment in pBlusescript. An R^s^P cassette was then added using the BspI201 and BamHI sites. The *R*^*s*^*P*_RRNA_ construct was then digested with SacI/AgeI and introduced into the 2T1 bloodstream-form *T. brucei* strain [10] that also contained a tetracycline-inducible I-SceI ORF introduced using the pRPa^i^ construct [17]. These Lister 427, clone 221a cells were grown and manipulated as described [10]. (B) A clonogenic assay to assess recovery from a DSB. Cells in all un-induced wells tested remained puromycin-resistant and cells in every induced well were puromycin-sensitive indicating loss of the *R*^*s*^*P* cassette in the latter case. Cell counts were carried out using a haemocytometer and tetracycline (used at 1 μg ml^−1^) was from Sigma. Data are derived from a pair of independent *R*^*s*^*P*_RRNA_ strains and error bars represent one standard deviation. (C) Physical monitoring of DNA resection adjacent to the lesion was carried out by slot-blot assay as described [1]. Genomic DNA samples were ‘native’, to detect ssDNA or denatured, to detect total DNA. The probes used on each blot are indicated on the right; the control probe is from chromosome 11 (Tb11.01.7240). Phoshorimager analysis was used to quantify the signals and ssRFP values were derived after correction for background, ssDNA versus total DNA and loading. The RFP ssDNA and total DNA plots indicate resection kinetics and DNA loss respectively.

**Fig. 2:**
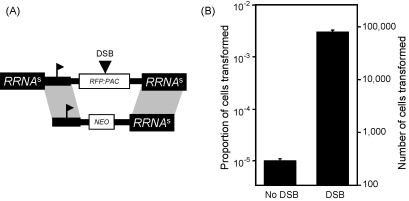
A genomic DSB increases transformation efficiency. (A) The schematic map illustrates the genomic target (reproduced from Fig. 1A) and the exogenous DNA construct. (B) Transformation assays. A DSB was induced by growth in tetracycline (1 μg ml^−1^) for 3 h. Nucleofection (Lonza) was carried out as described [7]. Briefly, 2.5 × 10^7^ *R*^*s*^*P*_RRNA_ cells were resuspended in 100 μl of human T-cell Nucleofector solution, mixed with 10 μg of purified linear DNA and subjected to Nucleofection using program X-001 in a 1 mm-gap cuvette. G418 was added <6 h later at 2 μg ml^−1^. To estimate the number of transformed clones, we initially used serial dilutions in 96-well plates but, due to concerns with loss of accuracy during extensive serial dilution, we used a modified approach to generate the data presented. Briefly, in duplicate experiments, 6 h after Nucleofection and drug addition, we distributed a sample predicted to contain 32 transformants (based on estimates from serial dilutions) over a 96-well plate. This approach yielded 15–40% positive wells per plate and was therefore deemed to have provided accurate scores. Error bars represent one standard deviation.

**Table 1:** Transformation efficiencies under different conditions.

Experiment^a^	Locus [sequence targeted]^b^	Construct [selectable marker]^c^	Time in Tet (h) [I-SceI/DSB-induction]	Electroporation method^d^	Transformation efficiency^e^
1	*Tb11.02.2110*	L [*BLA*]	–	E	<1 × 10^−7^
	[tubulin]	L	0	E	5 × 10^−7^
		L	3	E	3 × 10^−5^
		L	18	E	6 × 10^−6^
2	Tubulin (chr 1)	L [*BLA*]	0	E	6 × 10^−6^
		L	3	E	8 × 10^−5^
		L	18	E	1 × 10^−5^
3	*Tb11.02.2110*	C [*BLA*]	3	E	2 × 10^−6^
	[tubulin]	L	3	E	7 × 10^−5^
4	*RRNA*-spacer	L [*NPT*]	–	E	4 × 10^−7^
		L	3	E	8 × 10^−5^
5	*RRNA*-spacer (chr 2)	C [*HYG*]	–	N	<1 × 10^−7^
	[*hyg*/*RRNA*-spacer]	L^IH^	–	N	3 × 10^−7^
		L	–	N	1 × 10^−5^
6	*RRNA*-spacer	L [*NPT*]	–	N	1 × 10^−5^
	[*P*_*RRNA*_/*RRNA*-spacer]	L	3	N	3 × 10^−3^

a Strains and exogenous DNA used in each experiment: 1. Strain: *R*^*s*^*P*_2110_[1]; exogenous DNA: ptubBLAtub digested with XbaI/Bsp120I. 2. Strain: *R*^*s*^*P*_TUB_ (unpublished); exogenous DNA: As experiment 1. 3. As experiment 1. 4. Strain: *R*^*s*^*P*_RRNA_ (this manuscript); exogenous DNA: pbRn1 [18] digested with SacI/AgeI. 5. Strain: 2T1 [17]; exogenous DNA: pRPa^TAG^[17] digested with NgoMIV (LIH) or AscI (L). 6. Strain: *R*^*s*^*P*_RRNA_; exogenous DNA: pbRn1 digested with SacI/Bsp120I.

b Only shown if different to the locus. i.e. if the target was engineered at that locus. All targets are 200–600 bp in length.

c C, circular; L, linear with terminal targeting sequences; L^IH^, linear with internal targeting sequences; *BLA*, blasticidin deaminase; *NPT*, neomycin phosphotransferase; *HYG*, hygromycin phosphotransferase. A construct containing the *BLE* (phleomycin binding protein) gene yielded similar results in tests equivalent to experiment 6 (data not shown).

d E, standard electroporation (BioRad, Gene Pulser II); N, Nucleofection (Lonza). Approximately 7% [15] and 25% [7] of cells survive the procedure respectively.

e All values are the average of minimally duplicate experiments.
